# 2017 ISCB Overton Prize awarded to Christoph Bock

**DOI:** 10.1371/journal.pcbi.1005559

**Published:** 2017-06-30

**Authors:** Christiana N. Fogg, Diane E. Kovats, Bonnie Berger

**Affiliations:** 1 Freelance Science Writer, Kensington, Maryland, United States of America; 2 Department of Mathematics, MIT, Cambridge, Massachusetts, United States of America; 3 International Society for Computational Biology, Bethesda, Maryland, United States of America; 4 Computer Science and Artificial Intelligence Laboratory, MIT, Cambridge, Massachusetts, United States of America

**Figure:**
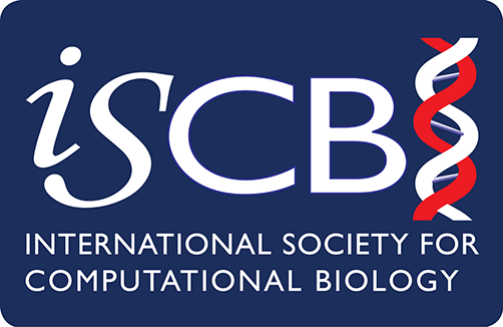


The International Society for Computational Biology (ISCB) each year recognizes the achievements of an early- to mid-career scientist with the Overton Prize. This prize honors the untimely death of Dr. G. Christian Overton, an admired computational biologist and founding ISCB board member. Winners of the Overton Prize are independent investigators who are in the early to middle phases of their careers and are selected because of their significant contributions to computational biology through research, teaching, and service.

ISCB is pleased to recognize Dr. Christoph Bock, principal investigator (PI) at the CeMM Research Center for Molecular Medicine of the Austrian Academy of Sciences in Vienna, Austria, as the 2017 winner of the Overton Prize. Bock will be presenting a keynote presentation at the 2017 International Conference on Intelligent Systems for Molecular Biology/European Conference on Computational Biology (ISMB/ECCB) in Prague, Czech Republic, which will be held July 21–25, 2017.

## Christoph Bock: At home in the epigenome

**Fig 1:**
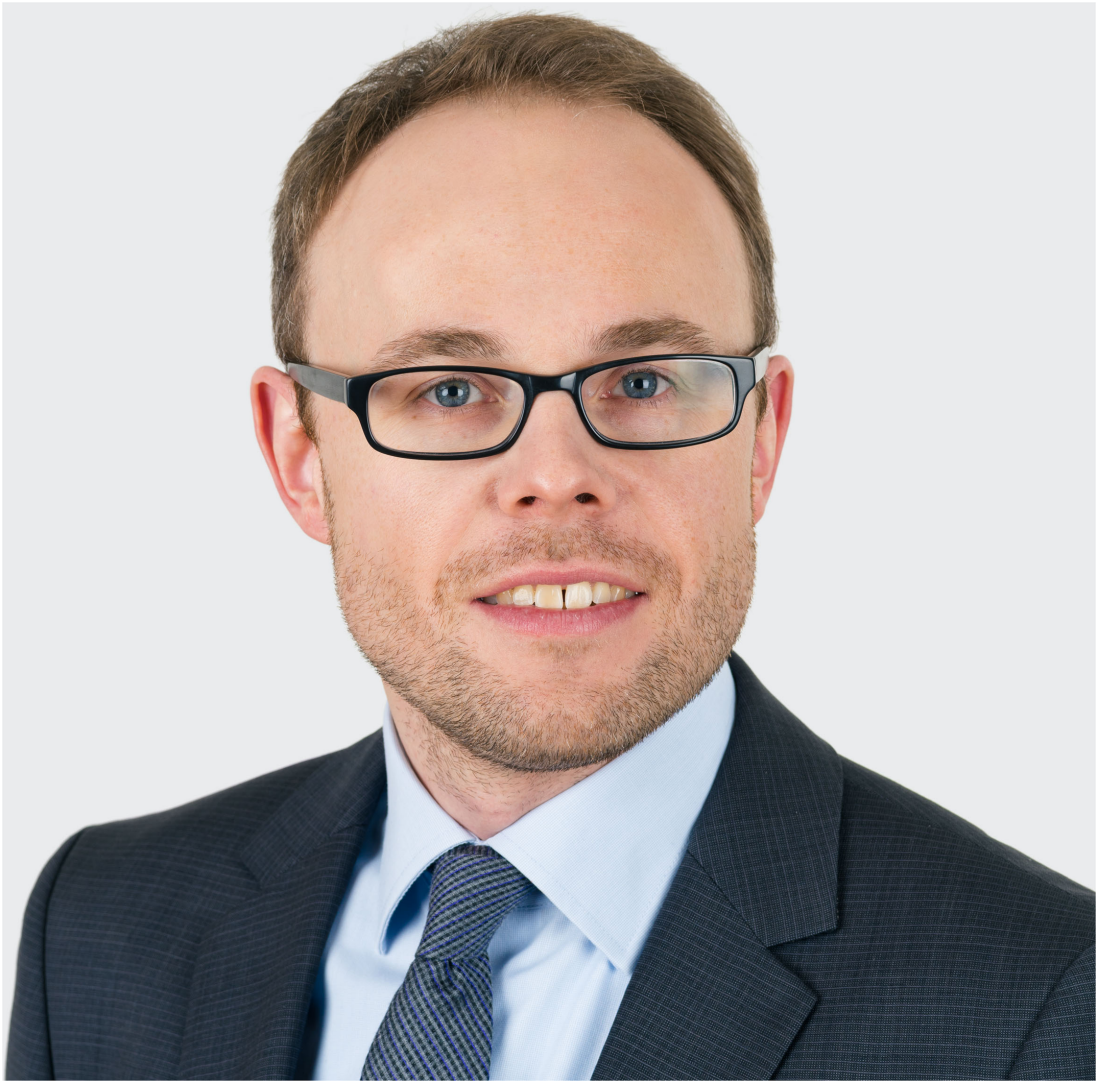
Christoph Bock.

Christoph Bock’s (Fig 1) scientific curiosity was nurtured from a young age. His parents were math and science teachers, and while they did not push him to pursue these areas of study, he sees how this intellectually stimulating environment cultivated his natural curiosity and provided a critical foundation to his career as a scientist. Bock started exploring computer programming from the age of 12, and he realizes in retrospect how learning to code was a valuable tool for practicing problem solving and scientific thinking.

During high school, Bock specialized in physics and math. His undergraduate studies at the University of Mannheim focused on computer science and business information systems, emphasizing machine learning and artificial intelligence. Toward the end of his studies, Bock yearned to tackle questions with broader relevance than the “toy problems” he encountered in his course work. Bock recalled, “Human biology seemed the biggest challenge and also most societally relevant. I was lucky that Jürgen Hesser offered a bioinformatics lecture and agreed to supervise my Master’s thesis at the University of Mannheim.” His Master’s research work focused on protein-structure prediction and homology modeling.

Bock pursued his PhD studies in bioinformatics under the supervision of Thomas Lengauer at the Max Planck Institute for Informatics, studying epigenetic regulation of the genome. “Moving into bioinformatics and epigenetics, I had to catch up on a lot of important biological knowledge,” Bock recalled. “Reading papers and collaborating was key, but it also helped that my research focused on a field that was quite young, with ample opportunity to try out something new.”

He attributes much of his bioinformatics training to the time spent in the research group of Thomas Lengauer, and he has been grateful for his mentor’s continued support and collaboration throughout his early career. Bock also acknowledges the important guidance and feedback on his research provided by Jörn Walter, who co-supervised his PhD dissertation and introduced Bock to the international epigenetics community.

Bock’s first encounter with epigenetics data transformed his scientific career path, and he has been one of the first bioinformaticians who dedicated their work to epigenetic data. He said, “When I started my PhD studies in 2004, the largest epigenetic dataset consisted of just over 100 data points, and one of my first papers established epigenome prediction as a means of inferring what was still very difficult and costly to measure experimentally.”

In the following years, next-generation sequencing transformed the field, and it became possible to collect several million data points in a single epigenome-mapping experiment. This development created a strong demand for bioinformatic methods. Bock said, “Working at the forefront of the epigenome revolution has been the highlight of my scientific research so far. But the most exciting times may still be ahead, as epigenome research is starting to become broadly relevant for medicine, and I am looking forward to contributing to this development.”

Bock developed several software tools as part of his PhD, including BiQ Analyzer for processing DNA-methylation data and EpiGRAPH for analyzing and predicting epigenome profiles in their genomic context. Bock went on to pursue postdoctoral studies under Alexander Meissner at the Broad Institute. There, Bock was exposed to the world of wet-lab biology, and he discovered the thrill and power of jointly developing new laboratory techniques and computational methods, which he used to study the epigenome of pluripotent and hematopoietic stem cells.

In 2012, Bock started his own research group at CeMM, an institute dedicated to advancing precision medicine through basic and translational research. He was hired by Giulio Superti-Furga, scientific director of CeMM, who, as Bock said, “provided ample encouragement and let me try things that were initially quite far outside of my comfort zone, such as starting a wet lab and leading a next-generation sequencing technology platform.” Bock has thrived at CeMM, where he has been able to work with many passionate researchers within the institute and at the neighboring Medical University of Vienna.

At CeMM, Bock has also developed his personal style of being a PI and mentor, acting as a catalyst of ideas and projects for an interdisciplinary team. He explained, “Our lab combines computational and wet-lab biology on roughly equal terms, with a good dose of technology development, including single-cell sequencing, CRISPR, epigenome editing, machine learning, and more. There is also an extensive network of collaborations, ranging from fundamental biology to immediate clinical applications in the area of personalized and precision medicine. It is a great privilege to work with such an interdisciplinary and creative group of smart people.”

Bock considers the success of his students and postdocs as a key measure of his achievement as a PI. He explained, “I work hard to maintain an environment in which every group member can build a great curriculum vitae (CV) and learns what he or she needs to advance in their scientific career. So far, we have a 100% success rate of postdocs moving on to attractive PI jobs, which is great for a young lab. But it is clear that helping others succeed in their career is not an easy task, and you need to create room for success and failure, and a safety net that encourages risk taking.”

Bock is still excited about epigenetics and what it can teach us about a cell’s past, present, and future. He hopes that epigenomic data can be used to understand the regulatory logic of cells and to determine what goes awry in diseases like cancer. Bock said, “We are pursuing an engineering-inspired ‘build it to understand it’ approach to cancer biology, where we combine CRISPR epigenome editing and computationally designed drug combinations to rationally reprogram normal cells into cancer cells and vice versa. Building upon a breakthrough technology for pooled CRISPR screening with single-cell sequencing, we seek to decipher complex biological pathways and gene regulatory networks in high throughput in order to overcome the classical ‘one gene, one postdoc’ paradigm of functional (epi)genomics.”

Bock is deeply gratified to be honored with the Overton Prize, especially since he will receive his award this year in Prague. He said, “Ten years ago, I attended ISMB 2007 in Vienna—one of the first conferences where I presented my PhD project on epigenome prediction. That year, Eran Segal won the Overton Prize, and his keynote lecture about DNA’s regulatory code reinforced my interest in understanding the role of epigenome regulation in biology and medicine. ISMB 2007 was also my first time in Vienna, and the great impressions from that visit surely contributed to the fact that a job ad from Vienna caught my attention a few years later. This year, it will be my pleasure to give the Overton Prize lecture at ISMB 2017 in Prague, 10 years and just a few hundred kilometers away from a truly career-defining ISMB 2007.”

